# Emerging mechanisms of lipid peroxidation in regulated cell death and its physiological implications

**DOI:** 10.1038/s41419-024-07244-x

**Published:** 2024-11-26

**Authors:** Yongxin Zheng, Junlu Sun, Zhiting Luo, Yimin Li, Yongbo Huang

**Affiliations:** 1 Department of Respiratory and Critical Care Medicine, The First Affiliated Hospital of Guangzhou Medical University, Guangzhou, China; 2 Guangzhou Institute of Respiratory Health Guangzhou, Guangzhou, China; 3 State Key Laboratory of Respiratory Diseases, Guangzhou, China; 4 Guangzhou National Laboratory, Guangzhou, China

**Keywords:** Cell death, Lipid signalling

## Abstract

Regulated cell death (RCD) refers to the form of cell death that can be regulated by various biomacromolecules. Each cell death modalities have their distinct morphological changes and molecular mechanisms. However, intense evidences suggest that lipid peroxidation can be the common feature that initiates and propagates the cell death. Excessive lipid peroxidation alters the property of membrane and further damage the proteins and nucleic acids, which is implicated in various human pathologies. Here, we firstly review the classical chain process of lipid peroxidation, and further clarify the current understanding of the myriad roles and molecular mechanisms of lipid peroxidation in various RCD types. We also discuss how lipid peroxidation involves in diseases and how such intimate association between lipid peroxidation-driven cell death and diseases can be leveraged to develop rational therapeutic strategies.

## Facts

- Lipid peroxidation can be considered a specific process of lipid metabolism.
- Excessive lipid peroxidation contributes to plasma membrane damage and further triggering multiple cell death modalities.
- Lipid peroxidation plays an important role in mediating interconnections between cell death pathways
- Lipid peroxidation inhibition (e.g., exogenous supplement with vitamin E or increasing GPX4 expression) represents a promising therapeutic option for several diseases by preventing cell death.

## Open question

- How does lipid metabolism impact the initiation, propagation and termination of lipid peroxidation.
- How does lipid peroxidation involve in regulation of various cell death patterns.
- What role does lipid peroxidation play in the interconnections of cell death?
- What is the physiology relevance of lipid peroxidation, and how can the promising preclinical studies be translated into effective clinical strategies.

## Introduction

**Fig. 1:**
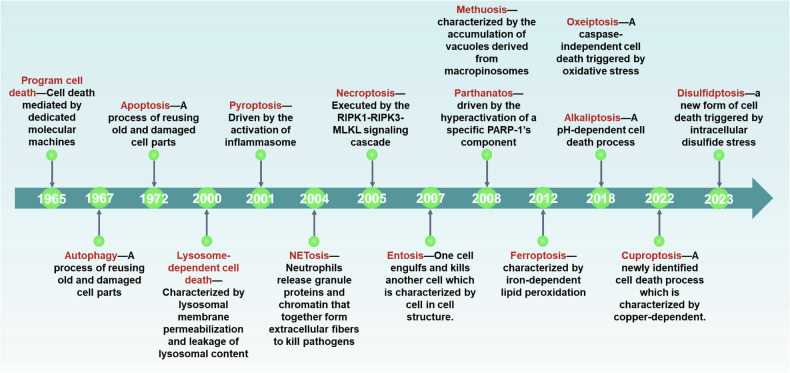
Timelines of terms used in various cell death modalities.

**Fig. 2:**
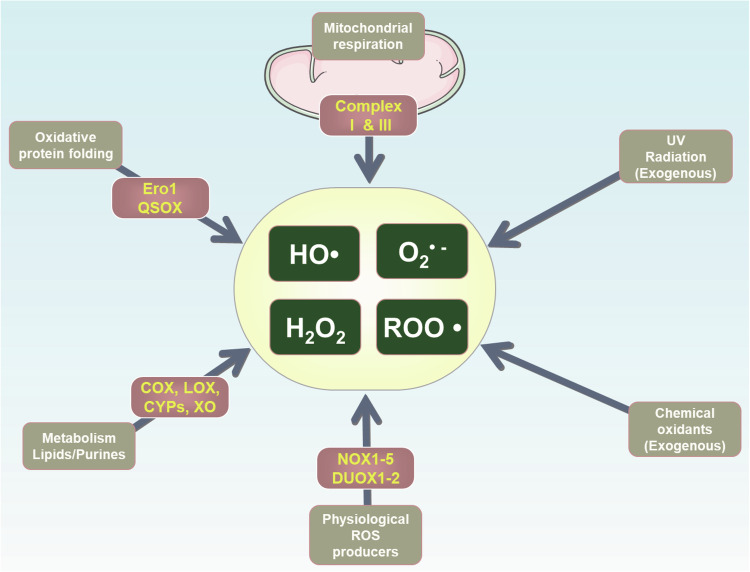
Reactive oxygen species (ROS) sources. ROS can be generated from various endogenous or exogenous sources. Ero1 endoplasmic reticulum-resident oxidoreductases, QSOX quiescin-sulfhydryl oxidase, COX cyclooxygenase, LOX lipoxygenase, CYPs cytochrome p450s, XO xanthine oxidase, NOX NADPH oxidase, DUOX dual oxidase.

Cell death is an essential component of organismal development and homeostasis which can be characterized by non-lytic and remarkably immunologically silent or lytic and pro-inflammatory [1]. Though the scientific observation of regulated cell death (RCD) historically began in 1842, the rapid growth in RCD research was only started since the term “apoptosis” was introduced in 1972 [2]. Since then, multiple RCD patterns have been identified (Fig. 1), each with unique regulatory mechanisms that influence physiological and pathological processes, including immune responses and tissue damage [3]. However, despite their difference, there are some common features among these cell death pathways. The excess production of reactive oxygen species (ROS) could be a predominant feature not only determined the cell death but also the cell death modalities [4]. ROS are some of the most common oxidants in cells, containing oxygen free radicals, e.g., hydroxyl radical (HO•), hydroperoxyl radical (ROO•), singlet oxygen, superoxide (O2-•) and hydrogen peroxide (H_2_O_2_) (Fig. 2). Increasing evidences implicates that the oxygen free radicals attack the plasma membrane (PM) lipids thereby altering the structure, activity and physical properties and finally lead to the production of lipid peroxides. If lipid peroxides, as a specific class of ROS, cannot be efficiently neutralized and subsequently accumulate in the PMs, might disrupt PM integrity and drive cells to death [5–7].

Excess lipid peroxidation of polyunsaturated fatty acid (PUFA) containing cell membrane phospholipids (PLs) is the crucial hallmark of ferroptosis. In the presence of bioactive iron, the PM-PLs can be converted to PL-hydroperoxide (PLOOH) through enzymatic or nonenzymatic lipid peroxidation mechanisms. The continued auto-oxidation of PLs is followed by increased curvature of membrane, thereby stimulating oxidative micellization, pore formation, and subsequent cell membrane damage [8]. Thus, aside from ferroptosis, some specific lipid peroxidation products have been demonstrated that contributes to the progression of various types of RCD (e.g., necroptosis, pyroptosis, apoptosis) [9–12]. This broader involvement of lipid peroxidation across various RCD types suggests that it act as a common mediator in these processes, although the precise mechanisms remain to be fully elucidated. Finally, a growing body of work has recognized lipid peroxides as key mediators of many cellular diseases and even death, indicating that decreasing the production of lipid peroxides might be an effective strategy to improve the lethal effects of lipid peroxidation [7, 13]. In this review, we will thoroughly discuss our current understanding of the myriad roles and molecular mechanisms of lipid peroxidation in various RCD types, and how lipid peroxidation involved in the regulating the pathogenesis of diseases, and emerging therapeutic applications of modulating lipid peroxidation.

## Lipid peroxidation

Lipid peroxidation is the process of generating lipid peroxide, where oxygen free radicals oxidize PUFAs in cell membrane and organelle membrane [14]. The chemical diversity and complexity of PLs are due to the combinations of two fatty acyl chains (sn1 and sn2). Typically, sn2 was tended to be occupied by PUFA, while sn1 was mainly attached by saturated fatty acids (SFA) and monounsaturated fatty acids (MUFA) [15]. Generally, PMs rich in SFA-PLs exhibit increased rigidity, which helps defend against lipid peroxidation and cell death. Conversely, PMs with higher degree of unsaturated fatty acids could be more pliable, and the presence of PUFA-PLs in the cell membranes is essential for fulfilling numerous cellular functions by increasing membrane fluidity. Interestingly, PUFA-PLs are particularly susceptible to peroxidation. With the oxygen free radicals or nonradical species attack, PUFAs will be oxidized to lipid peroxyl radicals and hydroperoxides, eventually lead to the cell membranes damage and cell death [16–18].

**Fig. 3:**
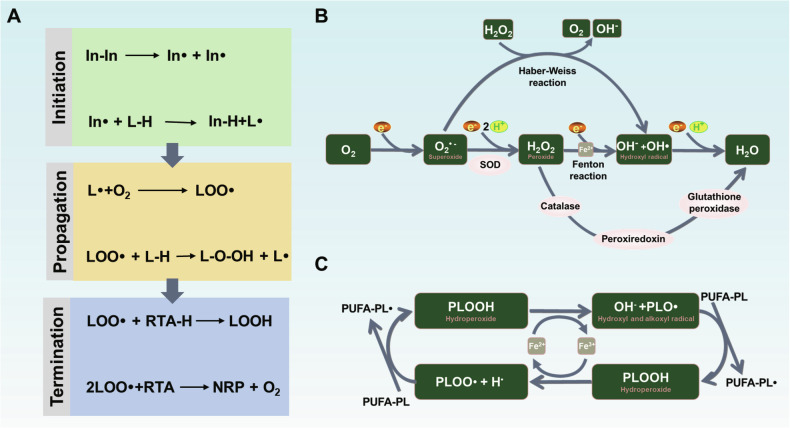
Lipid peroxidation. **A** The mechanism of free radical autoxidation understood by a nonenzymatic chain process consisting of initiation, propagation and termination. In the initiation phase, a lipid radical (L•) is generated by redox-active labile iron. In the propagation steps, molecular oxygen is transferred into the carbon-centered radical L• to generate a peroxyl radical (LOO•), and a hydrogen atom from the organic substrates is further added to LOO• to generate lipid hydroperoxide (LOO-H). The termination step is the transfer of an H-atom from antioxidants to remove radicals. **B** Molecular oxygen (O_2_) can be electrochemically reduced to water (H_2_O) via a 4e^–^ pathway, or hydrogen peroxide (H_2_O_2_) with 2e^–^ transferred in aqueous solutions. **C** The phospholipid hydroperoxides (PLOOH) can be used to generate radicals via Fenton reaction. The phospholipid radicals formed by this process can further react with other PUFAs to propagate the chain reaction of lipid peroxidation. In initiator, L lipid, L• lipid radicals, LOO• peroxyl radical, L-O-OH lipid hydroperoxide, RTA radical-trapping antioxidant, NRP non-radical products, PLO• phospholipid radicals, PUFA polyunsaturated fatty acid.

The lipid peroxide products of lipid peroxidation process have largely been understood [6, 19–21]. In the classical paradigm of lipid peroxidation can be understood by a chain process consisting of three steps: (1) initiation; (2) propagation; (3) termination. In the initiation step, the key event is the allylic hydrogen was abstracted to form the carbon-centered lipid radical (L•) [22]. Then, the propagation of the chain reaction occurs next when the molecular oxygen is added to the carbon-centered radical L• to generate a peroxyl radical (LOO•). The newly formed LOO• can subsequently abstracts the hydrogen from a second PUFA affords the lipid hydroperoxide (LOOH) [23]. Finally, the lipid peroxidation chain reaction will be broken when the LOO• encounters the radical-trapping antioxidant (RTA). The LOO• will abstract the hydrogen from RTA, initially generating a phenoxy radical, and in turn the newly formed products can couple with a second LOO• to yield a lipid hydroperoxide and other non-radical products (Fig. 3A) [24]. After the free radical chain reaction, PUFA are converted to their corresponding hydroperoxides.

## Initiation and propagation of lipid peroxidation

Lipid peroxidation can be initiated in cells by nonenzymatic and enzymatic mechanisms. The nonenzymatic lipid peroxidation is a process mediated by redox-active metals, especially iron. Within cells, labile iron is highly reactive which can react with the endogenously produced hydrogen peroxide (H_2_O_2_) to generate the hydroxyl radical (HO•). These redox reactions mediated by labile iron are known as “Fenton reaction”: Fe^2+^ + H_2_O_2_→ Fe^3+^ + HO• + OH^-^ [25]. With the Fenton reaction, both hydroxyl and peroxyl production can initiate the lipid peroxidation (Fig. 3B). Like H_2_O_2_, PLOOH can also undergo the Fenton reaction, a series of reaction where PLOOH can be converted to lipid hydroxy radical (PLO•) and lipid peroxyl radical (PLOO•). If PLOOH is not neutralized quickly, it can propagate the peroxidation to neighbor PUFA-PLs, which might further damage the PMs (Fig. 3C). Although the Fenton reaction has been well clarified in solution, its role in the cells and tissues is not thoroughly explored, and the reaction might only occur in severe iron overload states or other pathological states with iron metabolism disturbance [26]. Therefore, the processes resulting in the increase of cellular labile iron, such as uptake transferrin [27, 28], inhibition of the ferroportin exporter [29] and degradation of ferritin [30, 31], will sensitize the cell to lipid peroxidation and eventual cell death.

Multiple enzymes have been shown to drive lipid peroxidation. Cyclooxygenases (COXs), lipoxygenases (LOXs), and cytochrome p450s (CYPs) are the crucial enzymes that promoting the oxidized lipids production. COXs is the key enzyme regulating PUFA peroxidation. Upon catalysis of COXs, dihomo-γ-linolenic acid (DGLA) and arachidonic acid (AA) can be oxidized by radical-mediated lipid peroxidation and produce prostaglandins, which linking the lipid peroxidation directly to inflammation and apoptotic processes [32–34]. LOXs is a family of iron-dependent enzymes that are critical for the several lipid peroxidation pathways, can directly catalyze the formation of lipid radicals [35]. In humans, AA and linoleic acid are the most abundant PUFA that serve as the preferred oxidation site for LOXs using molecular oxygen to form hydroperoxyl radicals at different carbon position of acyl chains. ALOX5 converts AA into 5-hydroperoxyeicosatetraenoic acid (5-HETE), which can then be further transformed into leukotriene A4. Besides, ALOX12/15 has a unique substrate requirement that the ALOX12/15 possesses a greater scope than the ALOX5. PUFA (e.g., linoleic acid) can be oxidized to 13-hydroperoxyoctadecadienoic (13-HPODE) and 9-HPODE by ALOX12/15 [36]. To keep the broadened substrate scope, ALOX12/15 can be reactive towards intact PLs which do not require the PLs hydrolysis for peroxidation. Importantly, it should be noted that ALOXs is not the indispensable due to the existence of other enzymatic and nonenzymatic mechanisms for lipid peroxidation. Thus, the pharmacological inhibitors targeted ALOXs therapy should be used with caution, as many of them could neutralize with radicals and thus inhibit lipid peroxidation independently of ALOXs [35, 37]. P450 Oxidoreductase (POR), located in the endoplasmic reticulum (ER), plays a crucial role in initiating lipid peroxidation. The P450 enzymes is critical for the metabolism and detoxification of many drugs, steroids and many other types of chemicals via oxidation in ER [38]. Electrons donated by POR are crucial for CYPs, enabling these enzymes to oxidize and metabolize other molecules. CYP4A family, as the CYP family members, has been demonstrated to directly oxidize AA [39]. Recent studies have also reported that POR and CYB5R1 can transfer electrons from NAD(P)H to oxygen to generate the H_2_O_2_, which subsequently reacts with iron to generate hydroxyl radicals for further promoting the peroxidation of PUFA of PLs-PMs, thereby disrupting membrane integrity and leading to ferroptosis [40, 41]. Importantly, unlike the strictly regulated ALOX enzymes, POR is ubiquitously expressed, suggesting that POR might be a constitutive fashion for lipid peroxidation.

## Termination of lipid peroxidation

Significant advances have been made in understanding the enzymatic and nonenzymatic antioxidant systems within cells [42]. The most characteristic enzymatic system is the system xc^-^ -glutathione (GSH)-glutathione peroxidase (GPX4) axis, which represents the canonical mode of ferroptosis surveillance. The classical ferroptosis inducers, Erastin and RSL3, can trigger lipid peroxidation by inhibiting system x_c_^-^ and GPX4. System x_c_^−^ play a crucial role maintaining the intracellular GSH content by regulating the uptake of cystine into cells. GSH act as a cofactor for several antioxidant enzymes, especially GPX4. GPX4 can reduce the toxic hydroperoxides to nontoxic alcohols in biological membrane, which has an important role in defending lipid peroxidation [43–45]. Inhibition of GPX4 enzymatic activity, either through directly damage to the selenocysteine site or indirectly by suppressing system xc-, leads to reduced GSH levels and the propagation of lipid peroxidation. GPX4 is an essential protective gene that systematic GPX4 knockout is embryonic lethal in mice [46].

Besides GPX4, RTA is also an antioxidant system that can reduce PLOOH and thereby terminate the propagation of lipid peroxidation. Three GPX4-independent systems, including Ferroptosis suppressor protein 1 (FSP1)/CoQ10, dihydroorotate dehydrogenase (DHODH) and GTP cyclohydrolase 1 (GCH1)/tetrahydrobiopterin (BH_4_), has been identified in the last few years [47]. CoQ10 is an essential antioxidant involved in mitochondrial bioenergetics for its ability to carry two electrons, which can directly disrupt the lipid peroxidation chain and maintain the plasma membrane redox system. Importantly, CoQ10 exist in diverse membranes throughout cells, not just in mitochondrial, indicating that CoQ10 serves as a universal endogenous mechanism for protecting against lipid peroxidation [48]. FSP1 (also known as AIM2) functions to regenerate the ubiquinol form of CoQ10 through using NADPH. The FSP1-CoQ10-NADPH pathway could cooperate with GSH and GPX4 to scavenge lipid peroxidation intermediates [49, 50]. Similarly, DHODH can reduce oxidized CoQ10 by converting ubiquinone to ubiquinol within the mitochondrial inner membrane, thereby inhibiting ferroptosis [51]. GCH1 expression can inhibit lipid peroxidation through two mechanisms: (1) GCH1 can generate BH_4_, which functions to diminish endogenous oxidative radicals; (2) the increasing BH4 content can remodel the lipid membrane environment by increasing the abundance of reduced CoQ10 and depleting the PUFA-PLs [52, 53]. Interestingly, recent studies demonstrated that nitro oxide (NO) could also function as antioxidant to react with superoxide and generate reactive nitrogen oxides (RNS). Knockdown of nitric oxide synthases (NOS) will decrease the NO concentration which sensitized cell to ferroptosis [54, 55]. Recently, phospholipase A2 group VI (PLA2G6, also known as iPLA2β) was identified as an inhibitor of ferroptosis by hydrolyzing oxidized phosphatidylethanolamine [56–58], indicating that there are some other antioxidant mechanisms in addition to GPX4 and RTAs. These findings underscore the notion that an integrated antioxidant system protect against biological membrane damage caused by excessive lipid peroxidation.

## Lipid peroxidation in cell death

Recent studies have highlighted how specific lipid peroxidation products contribute distinctly to various types of RCD, such as ferroptosis, apoptosis, necroptosis, and pyroptosis. These findings suggest a complex interplay where lipid peroxides serve as not just passive byproducts but active mediators that modulate cell death pathways and influence disease pathogenesis. Understanding these interactions opens new avenues for targeted therapies that modulate lipid peroxidation to prevent or treat diseases characterized by aberrant cell death [59].

## Ferroptosis

**Fig. 4:**
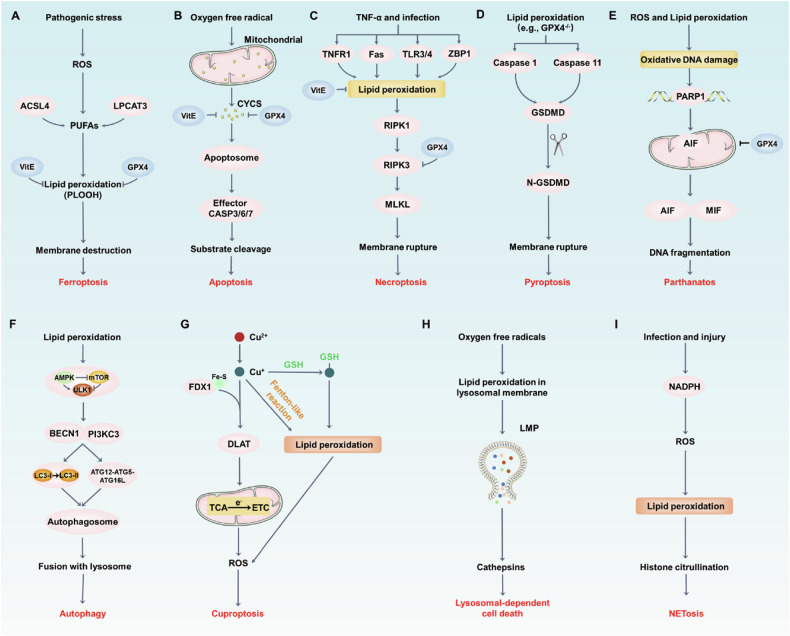
The core molecular mechanism of lipid peroxidation in regulated cell death. **A** The excess lipid peroxidation can activate the various cell death pathways, damage plasma membrane and eventually death. Exogenous supplement with Vitamin E (VitE) or the overexpression of GPX4 blocks cell death, including (**A**) Ferroptosis, (**B**) Apoptosis, (**C**) Necroptosis, (**D**) Pyroptosis, (**E**) Parthanatos, (**F**) Autophagy, (**G**) Cuproptosis. (**H**) Lysosomal-dependent cell death and (**I**) NETosis.

Currently, the most comprehensive understanding of the role of lipid peroxidation in cell death is through ferroptosis (Fig. 4A), a non-apoptotic form of RCD driven by iron-dependent lipid peroxidation [60]. This oxidative death is characterized by an accumulation of lipid peroxides that disrupts the integrity of the plasma membrane, ultimately leading to ferroptotic death [44, 47, 61]. The depletion of GSH or direct inhibition of SLC7A11-GPX4 pathway results in the accumulation of PLOOH and subsequently interacts with iron to form lipid radicals [46, 62–64]. The pharmacological induction of ferroptosis mainly targets SLC7A11-GPX4 pathway, such as erastin, RSL3, sorafenib and ML210 [65]. Besides, the lipid peroxidation process requires ACSL4 or LPCAT3 to mediate the reacylation of membrane phospholipids with PUFAs (e.g., AA) [16, 66]. Thus, for ferroptosis to proceed, PUFAs were required to intersect into membrane to serve as the targets for peroxidation and iron-catalyzed free radical production. Importantly, cells could be protected from ferroptosis by providing exogenous small molecule lipophilic antioxidants (e.g., ferrostatin-1, liproxstatin-1, trolox) and iron chelators (e.g., deferoxamine) that neutralize toxic lipid ROS or decrease the formation of lipid peroxyl radicals [60, 61, 67]. Notably, supplementation with the lipid antioxidant vitamin E (α-tocopherol) can effectively clear lipid peroxides and prevent ferroptosis [67]. Moreover, it enhances the cells’ ability to defend ferroptosis following GPX4 inactivation in vivo [68].

A major unsolved mystery is concerning how PUFAs peroxidation cause ferroptosis [69]. Although other types necrotic cell death such as pyroptosis and necroptosis are mediated by the formation of membrane pore, ferroptosis is believed to result from the accumulation of unrepaired cell damage [70]. Recent studies suggest that lipid peroxidation might create gaps in the plasma membrane, disrupting ionic homeostasis and leading to ferroptotic death without immediate rupture [70]. However, ninjurin-1 (NINJ1) -dependent lysis could be a driver following ferroptosis induction by promoting plasma membrane rupture and release of damage-associated molecular pattern molecules (DAMPs) during ferroptosis [71]. Besides, the generation of reactive products of lipid peroxidation might directly inactive intracellular proteins or trigger other pro-ferroptotic events [72, 73]. Although excess accumulation of lipid peroxides (4-HNE and MDA) has been shown to promote apoptosis and necrosis, the exact role in ferroptosis still remain elusive [74]. By solving these mysteries of ferroptosis, we may discover some novel therapies for ferroptosis-related diseases.

## Apoptosis

Similarly, numerous early studies also showed that lipid free radical act as the pro-apoptotic factors to activate intrinsic and extrinsic apoptosis pathways (Fig. 4B). Apoptosis was originally described as a regulated cell death that conserved in multiple organisms. The essential feature of apoptosis is the release of cytochrome c from mitochondrial, regulated by the balance of between pro-apoptotic and antiapoptotic proteins in BCL-2 family, activation of initiator caspases (caspase 8, 9, and 10) and effector caspases (caspase 3, 6 and 7) [75]. Mechanistically, apoptosis is initiated through two primary pathways: the intrinsic and extrinsic pathways [76]. The intrinsic pathway is activated by the oligomerization of the BCL2 family protein BAK and BAX, which contributing to the formation of oligomers from pores in the mitochondrial outer membranes, leading to the release of cytochrome c into the cytosol. Cytochrome c (CYCS), in conjunction with Apaf-1, forms the apoptosome in dATP/ATP-dependent manner, further recruiting procaspase 9 to initiate the caspase-processing cascade [77]. The extrinsic apoptosis pathway is activated by binding the death factors of the TNF family (such as FAS [Fas cell surface death receptor], TRAIL [TNF-related apoptosis-inducing ligand]), leading to caspase 8 activation. Finally, intrinsic and extrinsic pathways can activate the common effector caspases (e.g., 3 or 7) to trigger apoptosis [77].

During apoptosis, BAK and BAX-inducing lipidic pores in mitochondrial membrane are formed by fusion of the inner and outer lipid leaflets, indicating that the change of mitochondrial membrane is tightly associated with apoptosis. Lipid peroxidation is a crucial biochemical process that significantly influences the initiation and regulation of apoptosis, particularly through the oxidative modification of cellular membranes and the generation of reactive byproducts. The interaction between lipid peroxidation and apoptosis primarily occurs in mitochondrial, where critical signaling pathways converge to regulate cell death. One significant discovery is that cardiolipin (Cl) involved in apoptosis, a unique phospholipid located on the inner mitochondrial membrane. The peroxidation of CL plays a crucial role in promoting mitochondrial membrane permeabilization and enabling the release of CYCS, which then activate downstream caspases to execute apoptosis [78–80]. Besides, lipid ROS destroys the cellular membrane and mitochondrial membrane by oxidizing PUFAs, causing the formation of membrane pore and further resulting in the release of CYCS [81, 82]. Of note, 4-hydroxynonenal (4-HNE), as the end-product of lipid peroxidation, could increase the expression of Fas in a concentration- and time-dependent manner, accompanied by the activation of caspase 3 and finally leading to apoptosis [83]. This lipid peroxidation product can also alter intracellular calcium levels and mitochondrial membrane potential, further promoting the intrinsic pathway of apoptosis [42]. Furthermore, recent studies have found that the activation of LOX or ACSL4 can catalyze the oxidation of PUFA, leading to the accumulation of lipid peroxides and promoting apoptosis in cancer cells [84]. However, the precise mechanisms by which LOX and ACSL4 influence apoptosis remain complex and may involve both enzymatic and nonenzymatic pathways of lipid oxidation.

Similar to ferroptosis, GPX4 has also been recognized as an antiapoptotic factor both in vitro and in vivo [85–87]. Mechanistically, the overexpression of GPX4 protects cells from apoptosis by detoxifying the oxidative damage of membrane lipids, inhibiting mitochondrial release of CYCS, and inactivating caspase 3 [87]. In vivo, mitochondrial GPX4 (mGPX4) is essential for the maturation of cone photoreceptors, where the mGPX4 knockout mice exhibit a cone-rod dystrophy-like phenotype due to apoptosis [88]. Vitamin E supplementation significantly ameliorated photoreceptor loss, indicating that lipid peroxidation is tightly associated with the development of apoptosis [88]. Functionally, GPX4 ablation results in the failure of embryonic brain development, testicular dysfunction and short life span due to the increased apoptosis [89–92]. These studies emphasized the important role of lipid peroxidation in driving caspases-dependent and -independent apoptosis. However, it is still uncertain whether GPX4 exerts its antiapoptotic effect by directly inhibiting the activation of apoptotic proteins or by eliminating lipid peroxidation products.

## Necroptosis

Necroptosis, a programmed form of RCD showing features similar to apoptosis and necrosis, was discovered in studies of TNF (tumor necrosis factor) signaling with caspases inhibition [3, 93]. Briefly, when either caspase 8 or fas-associated via death domain (FADD) is depleted or the function is inhibited, TNF can induce caspase 8 independent necrosis through activating RIPK1 (receptor interacting serine/threonine kinase 1) [94]. RIPK1 subsequently interacts with RIPK3 to form the necrosome, a key signaling complex that activates the executor kinase MLKL [95, 96]. Finally, phosphorylated MLKL oligomerizes and translocates to membrane to elicit cell lysis, which eventually leading to the release of intracellular content and necroptotic cell death [75, 96, 97]. Currently, except for the activation of death receptor (e.g., FAS and TNFR1), necroptosis can be triggered by multiple stimuli, including activation of Toll-like receptor (TLR) 3 [98], TLR4 [99], nucleic acid sensors (e.g., Z-DNA-binding protein 1, ZBP1, also known as DNA-dependent activator of interferon-regulatory factor (DAI)) [100–102]. TLR3 is activated by double-stranded DNA in the endosomal compartment, whereas TLR4 can sense the extracellular bacterial lipopolysaccharide (LPS) and then transport to the endosomal compartment. ZBP1 is an emerging innate sensor of Z-form nucleic acids, which has the left-handed double-helical structure to identify the virus RNA (e.g., influenza A virus, SARS-CoV-2) [102, 103]. The activated ZBP1 can interact with the RIP homotypic interaction motif (RHIM) domain of both RIPK1 and RIPK3 to mediate the NF-κB activation and necroptotic cell death [100, 102, 104]. While various stimuli can induce necroptosis, the process is tightly regulated by lipid peroxidation, especially mitochondrial ROS. ROS generated by mitochondria, particularly in response to cellular stress, act as signaling molecules that amplify necroptosis through promoting RIPK1/RIPK3/MLKL activation.

In the structure, different residues in the C-terminal pseudokinase domain of MLKL (S345/S347/T349 in mouse and S357/T358 in human) are phosphorylated by RIPK3, which induces a conformational change and promote the binding of inositol hexaphosphate (IP6) with positively charged patches in the N-terminal domain of MLKL [105–107]. Subsequently, MLKL is recruited to phosphatidylinositides and inserted into the plasma membrane, suggesting that lipid composition and phosphorylation status in membrane lipid are the critical mediators of necroptosis [108, 109] (Fig. 4C). Recently, several studies have showed that promoting the synthesis of very long chain fatty acids or exogenous supplementation of PUFA enhance necroptosis under oxidative stress [110, 111]. These evidences emphasized that membrane-related lipid changes play a direct role in regulating necroptosis. Moreover, lipid peroxides interact with the membrane are essential for necroptosis execution that promoting MLKL membrane insertion and pore formation. The chronic accumulation of lipid peroxides (e.g., 4-HNE) also induce necroptosis in retinal pigment epithelial (RPE) cells. Neccrostatin-1 (Nec-1), the necroptosis inhibitor by targeting RIPK1, can inhibit RPE cell death by inhibiting both lipid ROS accumulation, providing further evidence that lipid peroxidation drives the occurrence of necroptosis [73]. Notably, the first evidence of lipid peroxidation in necroptosis was demonstrated by developing a transgenic anemia animal model using GPX4 depletion [10]. Absence of GPX4 leads to functional activation of RIPK3, resulting in necroptosis but not ferroptosis in erythroid precursor cells [10]. Rescue experiments reveal that vitamin E is essential for preventing necroptotic cell death and suppressing anemia caused by GPX4 deletion [10]. Indeed, the role of different subcellular sources of lipid ROS in necroptosis have been described, especially mitochondrial. The overactivation of mitochondrial by the mitochondrial respiratory chain complex I BAY87-2243(BAY) remarkably increases cellular lipid ROS levels and further trigger necroptosis [112]. Overexpression of GPX4 will prevent BAY-induced cell death, while GPX4 knockdown potentiated cell death by increasing the accumulation of lipid products [112]. Interestingly, increasing redox-active iron and lipid peroxidation play an important role in the TNF-α-induced the formation of necrosome, suggesting intricate connections exists between ferroptosis and necroptosis [113–115]. Antioxidants, as well as iron chelators, were able to rescue L929 cells from TNF-α-induced necroptosis, implying that Fenton reaction involved in the execution of cell death [113]. Thus, these findings raise additional about how dysfunctional mitochondrial induces caspases-independent necroptosis and to what extent these different cell death pathways overlap or are interchangeable. Of importance, additional work is required to elucidate the mechanisms of lipid peroxides triggering necroptosis and upstream kinases involved in this process.

## Pyroptosis

Pyroptosis and necroptosis share similarities as inflammatory forms of cell death, both involving membrane pore formation and the release of intracellular contents, suggesting a connection between lipid metabolism and pyroptosis. Unlike necroptosis, pyroptosis is triggered by gasdermin pores in the plasma membrane [76]. It emerged as a distinct cell death program due to the activation of caspase 1 and caspase 11-dependent inflammasomes in macrophages infected with *Salmonella* [116, 117]. Next, the cleavage of GSDMD (gasdermin D) by caspase 1 and caspase 11 to produce the N-terminal fragment of GSDMD (N-GSDMD) is a critical event in the lethal cascade driving infection-associated pyroptotic cell death [118, 119]. Increased N-GSDMD was translocated into lipids on the plasma membrane, and further promoted cell lysis by oligomerizing and forming pores [120, 121]. The GSDMD pores in the membrane will collapse ion gradients and lead to an influx of water, ultimately resulting in ninjurin 1 (NINJ1)-dependent cell membrane rupture and the release of DAMPs [122]. Notably, small pro-inflammatory DAMPs, including IL-1β and IL-18, can directly exit the cell through GSDMD pores [123]. Hence, there may be a tight connection between plasma membrane lipids and pyroptosis.

Lipid peroxidation plays a multifaceted role in regulating pyroptosis, as it is involved in both promoting and inhibiting this pyroptotic pathway under different physiological and pathological conditions. Intense studies showed that cytoplasmic membrane lipid peroxidation could remarkably promote pyroptotic cell death [7, 124] (Fig. 4D). Mechanistically, the oxidative modification of phospholipids in the plasma membrane promotes structural changes that facilitate GSDMD pore formation, which eventually leads to cell swelling and lysis. Enhanced lipid peroxidation upon GPX4 deletion (GPX4^Mye−/−^) was shown to increase the expression of caspase 11 and N-GSDMD in response to LPS transfection and *E-coli* infection [12]. Knockout of caspase 11 confers protection in septic GPX4^Mye−/−^ mice. Interestingly, Tang’s study further showed that only lipid peroxides produced by the oxidation of phospholipids can induce pyroptosis [12]. PLCγ1, as signal transducer by hydrolyzing membrane lipid to generate second messengers, can promote calcium influx and mtROS production, which triggers the activation of NLRP3 and caspase 1 [12, 125]. In line with this, inhibition of lipid peroxidation by ALOX5 limits the activation of caspase 11 and pyroptosis in macrophages, which provides a potential therapy for treating sepsis [11, 126]. Similarly, fatty acid oxidation (FAO), the main pathway of fatty acid catabolism, promotes the activation of NLRP3 through NADPH oxidase 4 (NOX4). NOX4, acting as a source of cellular superoxide anions, not only enhances the expression of CPT1A but also increases the production of mtROS and self-derived ROS, ultimately activating the NLRP3 inflammasome [127, 128]. Thus, it is believed that NOX4-derived mtROS may stimulate NLRP3 by activating CPT1A-meidated FAO, indicating that lipid peroxidation plays a broad role in enhancing pyroptosis. Paradoxically, a recent interesting event was found that direct treatment with specific lipid peroxides (e.g., 4-HNE) at physiological concentrations inhibited the NLRP3 activation and macrophage pyroptosis [129]. The novel mechanism by which 4-HNE inhibits NLRP3 is through its ability to directly bind to the critical domains of NLRP3, disrupting its interaction with the adapter protein NEK7, a key step in inflammasome activation, thereby preventing caspase-1 activation. Based on these contradictory events, whether differences in the rate of lipid peroxide production affect the role of lipid peroxidation in inducing pyroptosis remains unresolved. Rapidly increasing lipid peroxides may inhibit pyroptosis, while chronic accumulation, such as in GPX4 deficiency, triggers inflammasome activation and cell death.

## Parthanatos

Parthanatos is a poly (ADP-Ribose) polymerase 1 (PARP1)-dependent RCD that is triggered by excessive oxidative stress-induced DNA damage and chromatolysis [130, 131]. Although there are some of same characteristics as other cell death patterns, parthanatos is a distinct RCD that is characterized by chromatin degradation and particle release into extracellular space. Compared with apoptosis, pathanatotic cell death occurs without the formation of DNA fragments and apoptotic bodies [132]. Furthermore, unlike necroptosis and pyroptosis, parthanatos occurs in the absence of cell swelling and lysis, but accompanied by plasma membrane rupture [133, 134]. Mechanistically, excessive DNA damage is induced by multiple pathological processes such as the accumulation of RNS, inflammatory injury and ROS-induced injury [134]. PAPR1 can recognize DNA breaks and utilize nicotinamide adenine dinucleotide (NAD+) and ATP to trigger extensive formation of poly(ADP-ribose)(PAR)polymer. Excessive PAR polymers are the major cell death signal in parthanatos [135, 136]. Apoptosis-inducing factor mitochondrial-associated 1 (AIFM1, also known as AIF) is also required for the parthanatos execution [137]. When mitochondrial membrane is depolarized, hyperactive PARP1 binds to AIF, prompting its translocation from the mitochondrial to the nucleus [138]. Interestingly, despite the activation of caspases in late stages of parthanatos, caspase inhibitors cannot inhibit the parthanatos process except for blocking PARP1 activity, confirming that PARP1-mediated cell death is caspase-independent [137, 139]. Subsequently, nucleus translocation of PARP1-dependent AIF interacts with macrophage migration inhibitory factor (MIF) to format the AIF-MIF complex, which further induces the DNA breakage and ultimately results in parthanatos [140]. Therefore, the two crucial mechanisms are that the transduction of PAR polymer to mitochondrial and the transfer of AIF from mitochondrial to nucleus to degrade chromatin.

Recent studies reveal that excessive lipid ROS accumulation and oxidative DNA damage are the causative factor causing aberrant parthanatotic cell death (Fig. 4E). The accumulation of lipid peroxides has been shown to exacerbate oxidative stress, thereby amplifying the activation of PARP1 and contributing to mitochondrial dysfunction, further promoting the release of AIF and triggering the parthanatotic cell death process [141]. Interestingly, although lipid peroxidation amplifies PARP-1 activation, inhibiting PARP-1 can significantly reduce lipid peroxidation and maintain mitochondrial integrity, underscoring the link between PARP-1 overactivation and oxidative damage in promoting parthanatos [142, 143]. Currently, several methods that effectively inhibit the occurrence of parthanatos by clearing lipid peroxides have been confirmed, such as using α-tocopherol (α-TOC) and ALOX12/15 inhibitors [144]. Conversely, with the degradation of GPX4 using cyclophosphamide, AIF-dependent parthanatos was triggered to suppress tumor growth [145]. Most importantly, the degradation of GPX4 did not lead to the occurrence of lipid peroxidation-mediated ferroptosis [145]. These findings further emphasize the relationship between lipid peroxidation and parthanatos. GPX4 inactivation unravel an intrinsic pro-apoptotic cascade that linking lipid peroxidation, GPX4 and AIF-mediated cell death. More studies are needed to explore whether the GPX4-mediated activation of parthanatos is a widespread mechanism in other disease models, and how the lipid peroxidation involved in the regulation of parthanatos.

## Autophagy

Autophagy is an evolutionarily mechanism that is mainly responsible for removing damaged organelles and eliminating intracellular pathogens to maintain intracellular homeostasis [146]. There are multiple processes to proceed autophagy, including microautophagy, macroautophagy and chaperone-mediated autophagy [147]. In micro- and macroautophagy, the cytoplasmic phagophores that engulf various cargos is delivered to the lysosome or by an autophagosome, respectively, while in chaperone-mediated autophagy, targeted proteins binding with chaperone proteins are translocated across the lysosomal membrane [148]. The autolysosomes degrade the cargos and release the autophagic substrates into the cytosol to reuse [149]. The crucial mechanism of autophagy relies on the ATG (autophagy related) protein family, which ensure the proper conformation of the complex. The complex consists of ULK1 (Unc-51-like-autophagy-activating kinase), inhibition of mTOR and ATGs proteins, which allow its interaction with other molecules to generate the distinct membrane compartments [148, 150].

Lipid peroxidation and autophagy are closely linked processes, where lipid peroxidation can trigger autophagic responses, and autophagy regulates lipid peroxidation to maintain cellular homeostasis (Fig. 4F). On the one hand, ROS could initiate autophagy by inhibiting mTOR and further increasing the phosphorylation of ULK1 [151, 152]. This activation enhances the expression of autophagy markers such as LC3, BECN1 and p62, promoting the autophagic process [152–154]. Previous studies showed that unsaturated lipid peroxidation-derived aldehydes such as 4-HNE triggered autophagy in a JNK-dependent manner by activating the endoplasmic reticulum stress (ER) response. Pharmacological inhibition of JNK prevented 4-HNE-induced LC3-II formation, suggesting that 4-HNE triggered autophagic responses could be partly attributed to ER stress [155]. Similarly, the increased levels of 4-HNE not only promoted the LC3-I conversion to LC3-II but also enhanced the expression of BECN-1 [155–157]. Except for 4-HNE, increased MDA level was also in parallel with mTOR inhibition and ULK1 activation [157, 158], indicating that an increased level of lipid products is generally associated with the increased autophagy. Therefore, preventing the formation of lipid products by overexpressing GPX4 could notably inhibit autophagosome formation [159, 160]. However, it is noted that the effect of 4-HNE on autophagy was found to correlated with its concentration. The moderately elevated levels of 4-HNE (<30 μM) induced a more significant modification within autophagy-related proteins than strongly elevated 4-HNE levels (>45 μM) [161]. Moreover, several studies suggest that 4-HNE can adduct with LKB1 to inhibit the activity of AMPK and further activate the mTOR pathway, resulting the disabled autophagy initiation [147, 162, 163].

On the other hand, activated autophagy simultaneously decreases ROS generation through removal of damaged mitochondria and activation of antioxidant transcription factors (e.g., Nrf2) [164, 165]. Mitophagy is the selective degradation of mitochondrial through autophagy, which plays a crucial role in removing damaged mitochondrial and thereby decreasing the production of ROS. Despite the complex mechanisms of mitophagy, PINK-PRKN pathway is the major mechanism involved in the regulation of mitophagy [166]. PINK1, as a mitochondrial damage sensor, is activated by mild oxidative stress and further triggers mitophagy to remove ROS and prevent cellular damage [167]. Thus, it is not surprising that there is a feedback loop where ROS activates autophagy, and activated autophagy triggers the degradation of oxidized proteins and damaged organelles to reduce ROS levels. Further elucidation of the dynamic relationship between lipid peroxidation and autophagy is important for expanding our knowledge in understanding cell fate.

## Other cell death patterns

Unlike ferroptosis, Cuproptosis is another metal-driven cell death modality that is characterized by copper- and mitochondrial respiration-dependence. The copper ionophore elesclomol triggers Cuproptosis in cancer cells, which is linked to ferrodoxin-1 (FDX1) levels, elevated mitochondrial respiration rate, and copper availability [168, 169]. Copper serves as an essential enzyme cofactor in oxidative metabolism, anti-oxidant defenses and neurotransmitter synthesis [168] (Fig. 4G). Excessive copper binds selectively to lipoylated proteins involved in the tricarboxylic acid (TCA) cycle, leading to proteotoxic stress and cell death by increasing ROS [170, 171]. The increased oxidative stress from lipid peroxidation can, in turn, enhance copper ion accumulation in mitochondria, promoting Cuproptosis [172]. Thus, one emerging hypothesis is that that increasing copper can react with reductive GSH, which might result in the decreased FDX1 and increased lipid peroxidation, further promoting oxidative damage [173–175]. Importantly, the Fenton reaction based on the release of Mn ions and the inactivation of GPX4 accompanied by accumulation of lipid peroxidation and ROS that can further sensitize Cuproptosis [173–175]. More interestingly, exogenous copper can promote ferroptotic cell death, but not Cuproptosis, by directly binding to GPX4 and further increasing GPX4 degradation [176]. These findings highlight the complex interplay between metal ions, lipid peroxidation, and different cell death pathways, offering new insights into the role of metal stress in RCD.

Lysosome-dependent cell death (LCD) is a subroutine of RCD initiated by perturbations in intracellular homeostasis and demarcated by the release of hydrolytic enzymes into the cytosol following lysosomal membrane permeabilization (LMP) [3, 76, 177]. Biochemically, LCD proceeds upon LMP when cells are exposed to various lysosomotropic detergents (e.g., lipid metabolites (phosphatidic acid, sphingosine), ROS), including cathepsins [178–181] (Fig. 4H). This leakage may further amplify or initiate other cell death signaling pathways, such as apoptosis, necroptosis [178–181], and ferroptosis. However, the upstream molecular mechanisms of triggering LMP are still elusive. Commonly, ROS play a prominent causal role in LMP that H_2_O_2_-driven luminal production of hydroxyl radicals by Fenton reaction leads to the destabilization of lysosome membrane upon massive peroxidation of membrane lipids [179, 180]. In the highly acidic and reducing environment within lysosomes, ferric iron is reduced to ferrous iron. The diffused H_2_O_2_ can react with ferrous iron, resulting in the formation of highly reactive •OH radicals. These •OH radicals can further abstract electrons from unsaturated fatty acids (FAs), creating unstable lipid radicals that initiate a self-propagating chain reaction of lipid peroxidation, ultimately leading to lysosomal membrane rupture and leakage [182]. Therefore, a recent study showed that DC661, a dimeric form of chloroquine that inhibits palmitoyl-protein thioesterase 1 (PPT1), promoted lysosomal lipid peroxidation, resulting in LMP and cell death [183, 184]. N-acetylcysteine (NAC) was the only antioxidant which potently attenuated both lipid peroxidation-mediated LMP and cytotoxicity [183, 184]. Notably, lysosome could also promote the production of GSH and further suppress lethal lipid peroxidation by catalyzing the albumin [185]. Given that complexity and importance have been identified in lysosomal lipid peroxidation as a mediator of LCD [132], additional work are required to elucidate how lipid peroxidation affect the LMP, and synergistic or complementary functions involving these LCD pathways need to be further assessed.

NETosis is a form of regulated cell death (RCD) characterized by the release of net-like DNA-protein structures (NETs) in response to infection. Elevated NETs not only play a role in innate immunity by trapping and killing pathogen but also contributes to the release of DAMPs and further damages host cells [186, 187]. Despite being a dynamic process, the cellular and biophysical bases of NETosis are not well understood. Currently, multiple signals and steps including NADPH-mediated ROS production, activation of kinase signaling cascades, and the release and translocation of granular enzymes have been demonstrated to initiate netotic cell death [186, 188] (Fig. 4I). This is followed by PAD4-mediated histone citrullination, chromatin decondensation, and the formation of chromatin fibers [189]. ROS is essential for NETs formation and NETosis activation. Stimulation of neutrophils with PMA or bacteria leads to a rapid increase in ROS production, thereby triggering extensive DNA damage, which ultimately results in chromatin decondensation and NETosis [190]. Treatment with ROS scavengers or depletion of NADPH oxidase fail to initiate NETosis [191]. In parallel, various fatty acids can induce NETosis, with FAs triggering a rapid form of NETosis involving ROS production via both NADPH oxidase and mitochondria, as well as histone citrullination, and requiring Akt kinase, ERK and JNK kinases [192]. Electrophilic products of lipid peroxidation formed in the presence of ROS (e.g., Isolevuglandins (IsoLGs)) promote the activation of NETosis, which play a direct role in immune activation in hypertension [193]. Increased oxidized phospholipids (OxPL) enhance NETosis by binding to neutrophil PAF receptor, acting in parallel with to accelerate atherosclerosis and thrombosis [194]. More interestingly, in intestinal ischemia‒reperfusion (II/R) model, elevated NETs formation induced Fundc1 phosphorylation at Tyr18, which promotes excessive mitochondrial ROS generation and lipid peroxidation, resulting in endothelial ferroptosis [195]. Administration of Fer-1 or inhibition of PAD4 remarkably reduced intestinal endothelial ferroptosis, indicating that NETosis may precede ferroptosis under certain conditions [195]. Indeed, NETosis may occur in downstream of death signals that involved in other cell death modalities, such as ferroptosis, necroptosis and pyroptosis [195–198]. GSDMD not only trigger pyroptotic death but also involve in the induction of NETosis to exacerbate inflammation and digest pathogen [199, 200], suggesting a crosstalk between NETosis and pyroptosis.

## The crosstalk of various cell death patterns

**Fig. 5:**
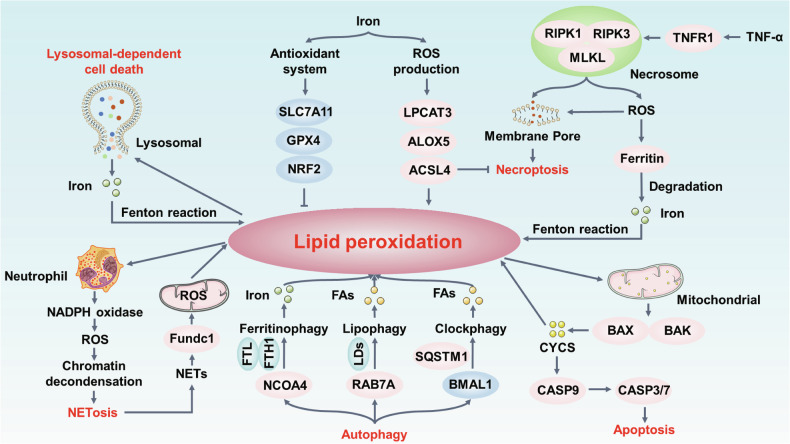
Emerging connectivity and crosstalk between lipid peroxidation and regulated cell death. Currently, a more complex network between the lipid peroxidation and different cell death pathways has been established over the years. Uncontrolled lipid peroxidation damages cellular membrane and lead to cell death. Then, the release of DAMPs from cell death might further exacerbate lipid peroxidation and trigger other cell death patterns.

As mentioned above, various forms of cell death were recognized as independent process with each relying on a different subset of proteins in initiation and execution of their respective pathways. However, a huge amount of evidence demonstrated that multiple cell death modalities are synergistically involved in the pathogenesis of disease [201]. Uncontrolled lipid peroxidation is not only a common signal involved in the initiation or execution phases of cell death, but also plays a crucial role in crosstalk of various cell death patterns (Fig. 5). In LCD, lysosome stores abundant reactive iron, indicating that the increasing LMP can result in the release of this toxic iron into cytosol [202]. This toxic metal will further amplify lipid peroxidation through the Fenton reaction, eventually contributing to ferroptosis [203, 204]. Thus, lipid peroxidation serves as a crucial link between LCD and ferroptosis, indicating that these two processes are tightly interconnected through iron-mediated oxidative stress.

Autophagy, as another degradation process, has shed light on the involvement of specific types of autophagy, especially ferritinophagy, lipophagy and clockohagy, in initiating or executing the ferroptotic process through the selective degradation of anti-damage proteins or organelles [205]. Ferritin is the major iron storage protein in most eukaryotic cells, consisting of ferritin heavy chain 1 (FTH1) and ferritin light chain (FTL). Previous studies demonstrated that autophagy contributed to ferroptosis by degradation of ferritin. The genetic suppression of ATG genes, including ATG3, ATG5, ATG7, ATG13 and ULK1, can limit erastin-induced ferroptosis with decreased intracellular Fe^2+^ levels and lipid peroxidation. Remarkably, nuclear receptor coactivator 4 (NCOA4), a key cytosolic autophagy receptor responsible for initiating ferritinophagy, mediates the degradation of ferritin, which subsequently increases intracellular Fe^2+^ levels and lipid peroxidation, ultimately leading to ferroptosis [206, 207]. In parallel, lipophagy also plays a role in promoting ferroptosis [208]. In ferroptotic cells, aggregation of lipid droplets (LDs) is observed in early stage which acting as a negative feedback loop to limit lipid peroxidation [209]. At the late stage, increased lipophagy releases PUFAs that serve as substrates for lipid peroxidation, whereas the knockdown of RAB7A or PGRMC1 inhibits lipophagy-induced ferroptosis [210, 211]. Clockphagy is an autophagic process that selectively degrades the core circadian clock protein aryl hydrocarbon receptor nuclear translocator-like (ARNTL/BMAL1), thereby promoting ferroptosis [212]. Mechanistically, the degradation of ARNTL/BMAL1 leads to the upregulation of EGLN2 (hypoxia-inducible factor 2) and subsequently suppressing the function of HIF1A (hypoxia-inducible factor 1 subunit alpha). Then, the inhibition of HIF1A further suppressing the expression of FABP3 (fatty acid binding 3) and FABP7, which ultimately promoting the occurrence of lipid peroxidation and ferroptosis [212]. Currently, clockphagy-induced ferroptosis is implicated in inflammation-related injury. The mice with specific deletion of pancreatic ARNTL/BMAL1 a are more susceptible to developing acute pancreatitis, and this pancreatic damage is inhibited by the administration of the ferroptosis inhibitor liproxstatin-1 [213]. Thus, dysregulated autophagy-dependent ferroptosis has implications for a diverse range of pathological conditions. These findings highlight the complex crosstalk between autophagy and ferroptosis.

The crosstalk between ferroptosis and necroptosis has also been extensively studied, with lipid peroxidation emerging as a key mediator of both pathways [10, 73, 214–216]. Lipid peroxidation have been considered essential downstream effectors of RIPK1-RIPK3 pathway, implying that ferroptosis might follow necroptosis [10]. TNF-α can dramatically stimulate ROS formation by favoring degradation of the ubiquitous iron-binding protein ferritin, leading to the increase reactive iron availability [115]. Subsequently, increased iron involves in the respiration chain and Fenton reaction that could result in the production of lipid ROS and eventually ferroptosis. Of importance, however, deletion of RIPK3 could not inhibit lipid peroxidation driven by GPX4 deletion, indicating that lipid peroxides can act as effectors of upstream of the necrosome independently TNF-α stimulation [10]. More interestingly, several studies support that ferroptosis and necroptosis are alternative pathways, such that resistance to one pathway sensitizes cells to death via the other pathway. Knockout of ACSL4 in ferroptosis-sensitive murine and human cells conferred protection from erastin- and RSL3-induced cell death, while loss of ACSL4 predisposed cells to necroptosis [214]. Altered lipid compositions of the membrane might be the key factor linking ferroptosis and necroptosis. MLKL drives basal resistance to ferroptosis by depleting PUFAs, whereas ACSL4 promotes basal resistance to necroptosis by making the membrane less susceptible to MLKL-driven permeabilization [16, 214]. These findings emphasize that lipd peroxidation is not merely a byproduct of cell death but is a crucial regulatory signal that facilitates crosstalk between necroptosis and ferroptosis.

Lipid peroxidation also plays a pivotal role in regulating ferroptosis and apoptosis. The crosstalk between these pathways is complex, with lipid peroxidation acting as mediator in their interconversion. Several studies have showed that apoptotic cell death can convert into ferroptosis under lipid peroxidation and that ferroptosis promote cellular susceptibility to apoptosis [217]. Under various stimuli (e.g., ROS), the permeability of the mitochondrial membrane increases, leading to the excessive release of pro-apoptotic factors (e.g., CYCS). CYCS further involved in Fenton-like reactions and lipid peroxidation, ultimately triggering ferroptosis [218]. Evolutionally, CYCS has been found to induce ROS-independent and cardiolipin-specific lipid peroxidation based on its redox state, indicating its specific role in lipid peroxidation [218]. Conversely, ferroptotic cell death also contribute to the apoptosis. Erastin, a known inducer of ferroptosis, can inhibit GSH synthesis and result in the accumulation of lipid peroxides and eventually cell death [219]. Notably, Erastin-induced GSH depletion also promotes cardiolipin peroxidation, which not only triggers the opening of the mitochondrial permeability transition pore but also induces caspase-dependent and -independent apoptosis [218, 220–222]. Excessive lipid peroxidation in ferroptosis will cause cytotoxicity and inflammatory reaction, which further induces apoptosis and other cell death patterns [216]. Ferroptosis inhibitors (e.g. liproxstatin-1, ferrostatin-1) can remarkably prevent apoptosis, indicating that ferroptosis occurs prior to apoptosis [216, 223]. These findings highlight the crosstalk between ferroptosis and apoptosis, encouraging further research to elucidate the regulatory mechanisms of the ferroptosis-apoptosis pathways.

## Lipid peroxidation in diseases and treatments

Recent studies have uncovered novel regulatory mechanisms at the intersection of lipid peroxidation and various RCD, offering insights into potential therapeutic targets. Controlling lipid peroxidation presents new opportunities for treating diseases characterized by dysregulated cell death, such as cancer and neurodegenerative disorders. Consequently, targeting lipid peroxidation is a valuable approach to improve the pathogenesis of diseases. In this section, we will discuss the potential physiological functions, and role of lipid peroxidation in diseases and therapy.

## Cancers

**Fig. 6:**
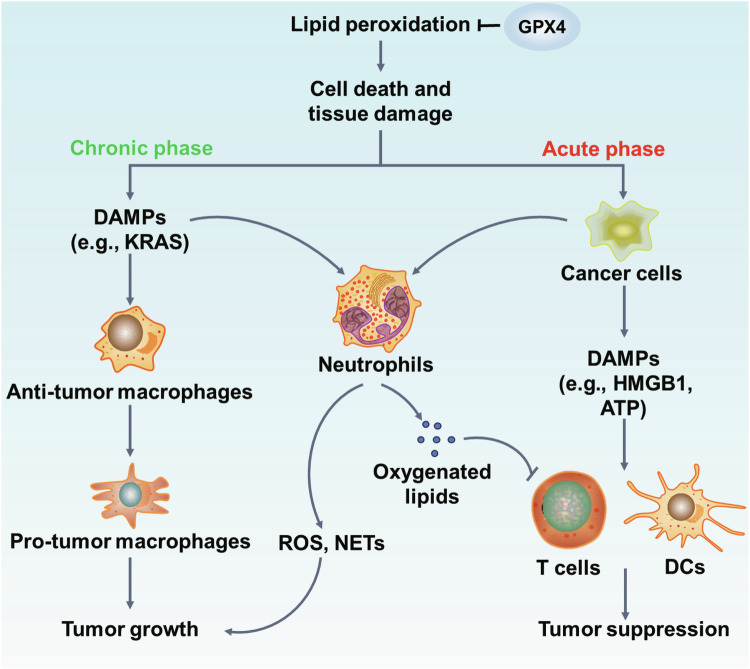
Dual role of lipid peroxidation in cancer. On the one hand, chronic inflammation triggered by lipid peroxidation may lead to the polarization the macrophage in tumor microenvironment, which further induce the release of DAMPs and promote tumor growth. On the other hand, the excessive tumor cells death via lipid peroxidation not only remove the malignant cells but also cause the release of DAMPs, lead to the accumulation of immune cells to suppress tumor growth. Neutrophils, as the important innate immune cells, also have an important role in regulating tumor growth.

Cancer cells are susceptible to perturbation of thiol metabolism, where excessive iron release and subsequent lipid peroxidation contribute to carcinogenesis [224]. Indeed, lipid peroxidation-driven cell death triggers tissue damage and chronic inflammation, which is the high risk to promote tumor growth. However, the induction of cell death is also the main principle for clearing tumor (Fig. 6). Agents that inhibit the cystine uptake via the cystine/glutamate antiporter (system x_c_^-^) (e.g., sulfasalazine) or directly deplete the GSH from plasma (e.g., cyst(e)inase) triggers ferroptosis and arrests tumorigenesis [224, 225]. Erastin were originally identified in phenotype screens for compounds that are selectively trigger engineered cancer cells death via lipid peroxidation [226]. Improved erastin analogs with increased solubility, selectivity and potency have been created, and further demonstrated the efficacy in clearing xenograft tumor [62]. Besides, numerous studies have showed that inhibition of GPX4 could increase the sensitivity of chemotherapy, radiotherapy, immunotherapy and nanotherapy by inducing lipid peroxidation and ferroptosis [227–229]. For example, RSL3, the specific inhibitor of GPX4, has been shown to inhibit the growth of fibrosarcoma in vivo [62]. Notably, altretamine, an FDA-approved anti-neoplastic drug that was applied in ovarian cancer treatment, enhances ferroptosis by inhibiting GPX4 [230]. However, current strategies not only triggers lipid peroxidation in malignant cells but also induces ferroptosis in immune cells simultaneously. Thus, several studies have developed some compounds to specifically induce ferroptosis in tumor cells, such as N6F11. N6F11 can specifically trigger the degradation of GPX4 in cancer cells without impacting the immune cells [231]. N6F11 treatment initiated HMGB1-dependent antitumor immunity mediated by CD8+ T cell which caused ferroptotic cancer cell death [231]. These findings may provide a safe and effective strategy to enhance ferroptosis-driven antitumor immunity, and the completion of clinical trials is needed to draw conclusion about the side effect.

Surprisingly, previous studies considered that ferroptotic cancers cells lack immunogenicity, which might impede the elicitation of properly processed and antigen presentation [232]. Co-culturing ferroptotic cancer cells with dendritic cells (DCs) reveals that ferroptotic cells decrease maturation of DCs and dampen antigen cross-presentation, and hence inhibit adaptive immune response and promote tumor growth [232]. Interestingly, CD8 + T toxic cells, the major executors of antitumor immunity, has been reported that ferroptosis in pathologically neutrophils induces the release of oxygenated lipids and further suppress the activity of T cells, which impede the antitumor immunity [232, 233]. Moreover, lipid peroxidation-driven plasma membrane rupture will accelerate KRAS-mediated pancreatic tumorigenesis by promoting DAMPs release, resulting in the activation of inflammatory pathways and polarization of macrophages for tumor growth [234]. Thus, though these studies are promising, more studies are needed to elucidate the association between lipid peroxidation and antitumor immunity before researchers can recommend different targeting therapeutic strategies.

## Neurological dysfunction

The growing body of evidence for the involvement of lipid peroxidation and general oxidative stress in neurodegeneration has generated much interest in using antioxidants as potential therapeutic approach. Alzheimer’s disease is characterized by the extracellular accumulation of amyloid-β (Aβ) that the insertion of Aβ into membrane will generate hydroperoxides, lipid peroxides and neurogenerative products (e.g., MDA) via Fenton reaction [235–237]. Adult mice with conditional knockout of GPX4 exhibited hippocampal neurodegeneration, while a ferroptosis inhibitor and vitamin E diet could significantly ameliorate neurodegeneration [238]. Currently, multiple clinical trials failed to observe the efficient of vitamin E and C in treating patients with neurodegenerative disease [239, 240]. Despite these disappointing results, antioxidants are still the promising therapeutics against neurodegenerative disease. Since the antioxidant capacity of a cell depends on a cocktail of multiple antioxidants and the time of initiating antioxidant therapy, researchers need to collect more data to draw the conclusion [241].

## Ischemia-reperfusion injury (IRI)

**Fig. 7:**
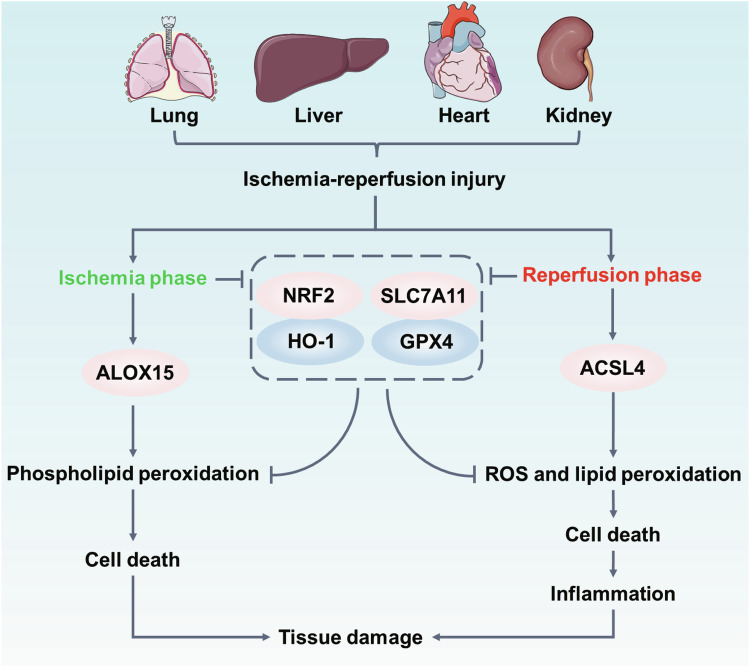
Lipid peroxidation in ischemia-reperfusion injury (IRI). In IRI, lipid peroxidation can simultaneously occur in ischemia- and reperfusion phase. The ischemia stage in IRI may promote the expression of ALOX15 to enhance phospholipid peroxidation, while the expression of ACLS4 is increased in reperfusion stage. Both enhanced lipid peroxidation will trigger cell death to further exacerbate tissue damage.

IRI followed by reperfusion can induce massive cell death and inflammatory response in affected organs, resulting in severe diseases including IRI-induced heart disease, kidney diseases, lung diseases and liver diseases [242]. Strong evidences support that ferroptosis is the major IRI-associated cell death due to excessive production of oxygen free radicals induced by IRI [243]. Mechanistically, reperfusion is responsible for excessive injury that the sudden reoxygenation accompanied by a burst of ROS inevitably exacerbates inflammatory response [244, 245]. The depletion of GPX4 can increase the sensitivity of hepatocytes or cardiomyocytes to lipid peroxides, which can accelerate IRI-induced liver or cardiac injury [246–248]. Similarly, given the sensitivity of ACSL4 as a promotor of lipid peroxidation, an initial study demonstrated that targeting inhibition of ACSL4 via dexmedetomidine protect kidneys against IRI-induced kidney injury [249] (Fig. 7). Activation of antioxidant system such as NRF2/HO-1 and SLC7A11/GPX4 pathways will be the effective therapies to preventing IRI damage by removing lipid peroxides [242]. Currently, more recent studies have suggested that reperfusion-induced lipid peroxidation depends on the critical events occurring ischemic stage [250, 251]. In IRI-induced cardiac injury, arachidonate 15-lipoxygenase (ALOX15) is regarded as the vital enzyme for the mediation of phospholipid peroxidation and ferroptosis in ischemic stage [18] (Fig. 7). Deletion of ALOX15 could obviously limit lipid peroxidation and increase GSH levels in cardiomyocytes subjected to ischemic [18]. Importantly, daidzein has been identified as a potent inhibitor that dramatically inhibited ALOX15 activity and held promise for developing natural medicines of cardioprotection [18]. Investigator must further evaluate the role of lipid peroxidation during different IRI stages.

## Infection

The activation of lipid peroxidation is implicated in pathogen-induced injury of host tissues. For example, *Mycobacterium tuberculosis* (Mtb) infection is associated with the reduced levels of GSH and GPX4, along with increased lipid peroxidation, which further lead to necrotic cell death. Using Fer-1 or overexpressing GPX4 are the effective treatments to prevent lipid peroxidation-driven cell death and decrease bacterial loads, implicating the GPX4/GSH axis as a critical target for treating Mtb infection [252, 253]. Moreover, as previously discussed, in a cecal ligation and puncture (CLP)-induced polymicrobial sepsis model, the conditional depletion of GPX4 triggers excessive lipid peroxidation and aggravates systemic inflammatory response that reportedly rely on GSDMD-dependent pyroptosis rather than ferroptosis [12]. Of note, Lipid peroxidation also exhibits activity to kill bacterial cells. The ability of AA to remove *S. aureus* is characterized by a lipid peroxidation mechanism where AA is oxidized into reactive electrophiles, which elicit toxicity by modifying the macromolecules of *S. aureus* [254]. Hence, modulation of lipid peroxidation at the host-pathogen interface is an attractive and exciting new area that it needs to further elucidate the specific role of lipid peroxidation in various pathogen infection.

**Fig. 8:**
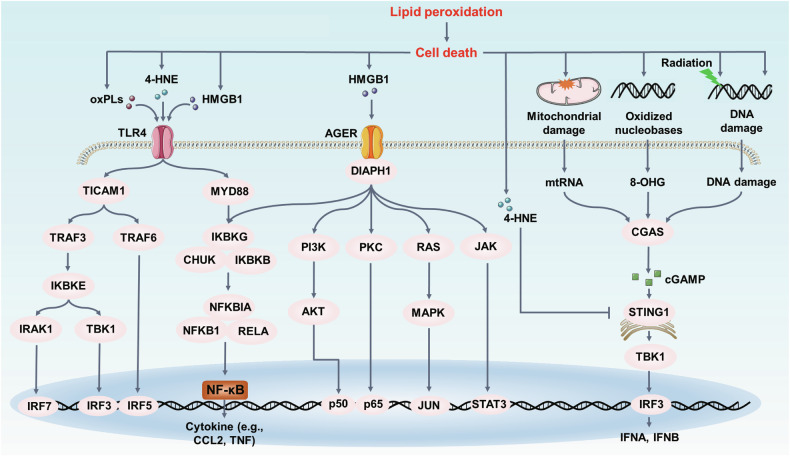
Lipid peroxidation in infection. Toll-like receptor 4 (TLR4) has an important role in innate immunity. The oxygenated lipids can activate TLR4 to further induce the activation of adapter TICAM1 or MYD88. Next, the activated TLR4 induces type I IFN signaling and NF-κB pathways to promote lipid peroxidation-related inflammation. Besides, AGER recognizes multiple DAMPs (e.g. HMGB1), leading to the activation of inflammatory signaling pathways, including phosphatidylinositol 3-kinase (PI3K)-protein kinase B, protein kinase C (PKC), RAS-MAPK and JAK-STAT. In STING1 pathways, lipid peroxidation can damage mitochondrial and DNA, whereas the release of mtDNA, 8-OHG and damaged DNA activates the STING1-dependent inflammatory pathway via the DNA sensor CGAS. However, lipid peroxides (e.g., 4-HNE) can directly inhibit STING1 activity by the carbonylation of STING1.

Lipid peroxidation also play an important role in antiviral innate immunity. In COVID-19, lipid peroxidation levels (MDA and 4-HNE) are the independent risk factors for 28-day intubation/death in COVID-19 patients, supporting the important role of lipid peroxidation in SARA-CoV-2 infection [255]. GPX4 is the essential enzyme for innate immune system by reducing oxidized molecules. A T cell-specific GPX4-deficient mice displays a phenotype of accumulated lipid peroxides, leading to an intrinsic defect in defending acute lymphocytic choriomeningitis virus (LCMV) and Leishmania major parasite infections [256]. Notably, GPX4-deficient CD4+ and CD8+ T cells fail to expand in the context of acute infections, which is rescued by supplementation of VitE [256]. Evolutionally, mTORC2-AKT-GSK3β axis was identified as a critical signaling hub to promote the longevity of CD4+ T cells by preventing LCMV-induced lipid peroxidation [257]. Additionally, multiple immune-related pathways were activated via lipid peroxidation, such as TLR4, AGER and STING1 pathways (Fig. 8). Among them, STING1 pathway, as the next-generation immunotherapy target in infection, is tightly associated with the lipid peroxidation. The depleted GPX4 followed increased lipid peroxidation limits the STING1-mediated type I IFN antiviral immune responses during herpes simplex virus 1 infection in mice [243, 258]. Mechanistically, increased 4-HNE inhibits STING1 activation by direct carbonylation in macrophage [259]. However, it is interesting that the release of oxidized nucleobases (e.g., 8-OHG) can activate the STING1-mediated inflammatory response in macrophages [259]. Importantly, hyperactivation of STING1 is also a bridge between autophagy and ferroptosis that STING1 directly promotes ferroptosis by overactivating autophagy in the context of DNA damage [260]. Thus, in the future, it will be important to evaluate the complex relationship between STING1 and ferroptosis. A comprehensive evaluation of the STING1-targeted drugs will be helpful for infectious diseases.

## The implication of targeting lipid peroxidation

Due to the important role of lipid peroxidation in many diseases and death, intense effort has been made to identify and develop compounds for targeting lipid peroxidation (Table S1). Based on the relationship between lipid peroxidation and cell death, molecules that increase production of peroxides to trigger cell death might be important for removing tumor, and molecules that prevent or eliminate peroxides might be important for defending inflammatory diseases.

## Increasing lipid peroxidation

Redox balance with abundant GSH is important for the longevity of tumor cells. Limiting the GSH formation by inhibiting system x_c_^-^ is a promising approach to kill tumor cells due to the excessive active metabolism and sustained oxidative stress [43]. Obviously, a growing body of preclinical evidences suggests that the induction of lipid peroxidation by inhibiting system x_c_^-^ might be an effective therapeutic strategy to clear cancer cells and prevent the acquired resistance to several cancer drugs, such as lapatinib, dabrafenib and vemurafenib (Table S1) [228, 261, 262]. Importantly, some ferroptosis inducers can also act synergistically with most traditional drugs (e.g., lapatinib) to suppress the tumor growth (Table S1) [263]. Hence, determining the sensitivity of tumors to system x_c_^-^ is required to apply system x_c_^-^ inhibition-based therapies. Besides, immune checkpoint blockade and radiotherapy as potential options also enhance lipid-peroxidizing activity and GSH-depleting activity to trigger tumor cells death [264, 265], indicating that lipid peroxidation is a promising and effective therapeutic strategy for suppressing tumor growth.

## Inhibiting lipid peroxidation

The most common strategy to prevent lipid peroxidation is inhibiting the activity of lipoxygenase enzymes. Among them, 5-lipoxygenase, as the crucial enzyme of driving lipid peroxidation, has been intensively studied with many inhibitors advancing into clinical studies [7, 266]. However, only Zileuton was approved for clinical therapy, which is believed to ligate the active iron through its N-hydroxy urea moiety [267]. Besides, the important role of other lipoxygenases such as 12- and 15-lipoxygenases in diseases have also been recognized, which promoting the development of specific inhibitors of these lipoxygenases [268, 269]. Currently, an emerging strategy is to administer PUFAs that incorporates deuterium at the bisallylic position [35, 270]. Such deuterated PUFAs exhibit chemical resistance to lipid peroxidation, which has shown to be an effective inhibitor of ferroptosis and various chronic degenerative diseases. Similarly, MUFAs can also inhibit lipid peroxidation, presenting them as potential therapeutic options [271].

## Eliminating lipid peroxides

Eliminating the peroxide or decreasing the radical intermediate is an effective strategy for preventing the production of lipid peroxides. GPX4, the primary reductant in biological system, is responsible for reducing lipid peroxides by using selenocysteine as a cofactor [272]. Dietary selenium can suppress the impact of loss of GPX4, implying that the diet of model organisms may be an important variable in influencing the susceptibility to lipid peroxidation [273]. Besides, vitamin E is another physiologically essential antioxidant able to mitigate the toxicity arising from lipid peroxides [274]. The supplementation with vitamin E can inhibit the increased lipid peroxidation by scavenging lipid peroxyl radicals to terminate chain propagation independent of the type of free radicals which initiating chain reaction [275]. However, the antioxidant effects of vitamin E depend on the nature of both oxidants and the substrates being oxidized. Vitamin E is believed to be the specific reductant of lipid peroxidation that it is unable to effectively scavenger other ROS such as hydroxyl radical [274, 275].

## Conclusion and perspectives

RCD occurs through a variety of subroutines that causes the decomposition of cells in different ways, hence various cell death modalities have distinct morphological changes and molecular mechanisms. However, lipid peroxidation can damage DNA, protein, lipids and eventually lead to cell death. Multiple protective mechanisms, including the antioxidant systems, are activated to scavenger oxygen free radicals and lipid peroxides. Hence, a comprehensive understanding the role and mechanisms of lipid peroxidation in cell death has revealed opportunities to regulate aberrant cell death in diseases. In recent years, we have witnessed substantial progress in lipid peroxidation research, including the understanding of the mechanisms of lipid peroxidation and pathological functions of this unique oxidative stress. Although lipid peroxidation and relative regulatory mechanisms have been established as crucial mediators of various RCD, many fundamental problems still remain elusive and will be the potential discoveries to be made in the future. First, we note a major challenge is that we do not know how lipid peroxidation ultimately leads to cell death. Uncontrolled peroxidation of PUFA-PLs is identified as the crucial downstream mechanism that it may damage membrane and lead to the pore formation, comprising the integrity of membrane. However, changing the components of membrane such as enriching PUFAs causes failure of some vital functions associated with membrane, resulting in cell death [35]. Besides, the lipid peroxides can directly damage other macromolecules, inactivate key structural and functional proteins within cells just like caspases serve in apoptosis. We hope the mechanisms of lipid peroxidation driving cell death will be explicitly elucidated in the future. Secondly, it is known that the process of cell death is an extremely complicated interconnected process involving multiple molecules. The intricate connections among various cell death pathways are confusing: is lipid peroxidation a direct cause of cell death, or does it initially induce one type of cell death and subsequently another [276, 277]. As mentioned above, the PLs peroxidation of membrane can lead to the leakage of cellular contents, and the release of DAMPs will further initiate other cell death ways. Third, tissue or cell selectivity for lipid peroxidation-driving cell death remain elusive. Such selective activation or inhibition in specific tissues, cells and disease contexts may be critical for developing more precisely targeting therapies. For example, increasing the production of lipid peroxides via targeting GPX4 may systematically cause toxicities and lead to organs damage, such as kidney injury and neurodegeneration. However, selectively inactivating GPX4 in tumor cells could be effective and may offer a safe therapeutic window. Currently, multiple means including drug delivery, optimized biodistribution and pharmacokinetics have been put forward to enhance the precision of therapies. Elucidating these strategies and potential mechanisms may be critical for leveraging the therapeutic advantages of lipid peroxidation to treat diseases. Finally and technically, there is an urgent need to develop an effective and convenient technology to accurately measure lipid peroxidation. Fluorescent probes (e.g., C11-BODIPY) widely used to detect lipid peroxidation but cannot distinguish the peroxidation of PLs or free fatty acids [5]. Oxylipidomics can identify specific categories of oxidized phospholipids, which helps to determine the mechanisms underlying the selectivity of peroxidation and the roles that these specific oxidized phospholipids play in cell death. Addressing these elusive questions not only provide new insights into lipid peroxidation-driving cell death, but also yields novel drug targets, biomarkers, and therapeutic strategies for diseases.

## Supplementary information

Table S1
