# Supplementary material for: Emerging mechanisms of lipid peroxidation in regulated cell death and its physiological implications
Source: Cell Death Dis. 2024 Nov 26;15(11):859. doi: 10.1038/s41419-024-07244-x (PMC11589755; doi:10.1038/s41419-024-07244-x)
Supplement: Supplementary file 1 — Table S1 [file 41419_2024_7244_MOESM1_ESM.docx]

Table S1. Summary of related drugs that may induce lipid peroxidation in multiple diseases.

| **Intervention** | **Target** | **Trial** | **Phase** | **Disease** | **Primary endpoint** | **Main results** |
| --- | --- | --- | --- | --- | --- | --- |
| Altretamine +  etoposide | GPX4 Inhibition | NCT00002936 | I | Lymphoma, Sarcoma | NA | NA |
| Artesunate | Increased free iron | NCT02633098 | II | Colorectal cancer | Recurrence free survival | Ongoing |
| Artesunate |  | NCT03093129 | II | Colorectal cancer | Recurrence free survival | Ongoing |
| Artesunate |  | NCT00764036 | I | Breast cancer | Safety | Up to 200 mg/d oral artesunate  were safe and well tolerated |
| BSO + melphalan | Glutamate–cysteine ligase inhibition | NCT00002730 | I | Neuroblastoma | NA | NA |
| BSO + melphalan |  | NCT00005835 | I | Neuroblastoma | Maximum tolerated dose | NA |
| BSO + melphalan |  | NCT00661336 | I | Neuroblastoma | Maximum tolerated dose | NA |
| Lapatinib | ferroportin-1 activation | NCT03085368 | II/III | Breast cancer | Overall survival | Ongoing |
| Lapatinib + taxane |  | NCT00667251 | III | Breast cancer | Disease-free survival | Ongoing |
| Lapatinib + paclitaxel |  | NCT00356811 | II | Breast cancer | Disease-free survival | First-line lapatinib plus paclitaxel produced an encouraging overall response rate with manageable toxicities |
| Neratinib + capecitabine | Increasing iron | NCT03377387 | I/II | Breast cancer | Maximum  tolerated dose | Ongoing |
| Neratinib |  | NCT04366713 | II | Breast cancer | Changes in colon Pathology | NA |
| Neratinib + trastuzumab / cetuximab |  | NCT03457896 | II | Colorectal cancer | Progression free survival | Ongoing |
| Sorafenib | SLC7A11 inhibition | NCT00064350 | II | Lung cancer | Complete response | The complete response was 0%. |
| Sorafenib |  | NCT02559778 | II | Neuroblastoma | Event free survival | Ongoing |
| Sorafenib + busulfan + fludarabine |  | NCT03247088 | I/II | Acute myeloid leukemia | Maximum tolerated dose | Ongoing |
| Cisplastin | Decreased GSH | NCT04574960 | III | Bladder cancer | Disease-free survival | Ongoing |
| Cisplastin |  | NCT01561586 | III | Cervical cancer | Overall survival | Ongoing |
| Cisplastin |  | NCT03649321 | I/II | Pancreatic cancer | Complete response | Ongoing |
| Fluvastatin | HMGCR | NCT00416403 | I | Breast cancer | Change in Ki-67 level | Proliferation (Ki-67 immunostaining) decreased by a median of 7.2% in high-grade tumors |
| Atorvastatin |  | NCT00816244 | II | Breast cancer | Changes in Ki-67 level | Atorvastatin may contribute to these anti-proliferative effects |
| Simvastatin |  | NCT03454529 | II | Breast cancer | Changes in Ki-67 level | Ongoing |
| Sulfasalazine | SLC7A11 inhibition | NCT04205357 |  | Glioblastoma | Toxicity | Ongoing |
| Sulfasalazine |  | NCT03847311 |  | Breast cancer | Overall pain relief | Ongoing |
| Sulfasalazine |  | NCT01577966 |  | Glioma | Change in glutamate levels | Decreased glutamate release. |
| Withaferin A | GPX4 inhibition | NCT00689195 | I/II | Osteosarcoma | Response, toxicity, disease progression | NA |
| Withaferin A |  | NCT04092647 | II | Breast cancer | Perceived cognitive impairment | Ongoing |
| CPX | Iron chelator | NCT00990587 | I | Relapsed or refractory hematologic malignancy | Toxicity, maximum tolerated dose | NA |
| CPX |  | NCT00382330 | I | Vulvar Cancer | Precancerous lesion(s) shrink | NA |
| Deferoxamine | Iron chelator | NCT00777140 | II | Ischemic stroke | Clinical and analytical adverse events | Increased body iron stores are associated with poor outcome, symptomatic hemorrhagic transformation, and severe edema in patients |
| Deferoxamine |  | NCT04633889 | II | Acute kidney injury | Acute kidney injury | Ongoing |
| Deferiprone |  | NCT05604131 | I | Acute myocardial infarction Type 1 | Treatment efficacy | Ongoing |
| Deferiprone |  | NCT00907283 | II | Neurodegeneration with brain iron accumulation | Efficacy, safety | Chelating treatment might be effective in improving neurological manifestations associated with iron accumulation. |
| Deferiprone |  | NCT05111821 | II | Stroke | R2* Index | Ongoing |
| Acetaminophen | System x_c_^-^ inhibition | NCT04291508 | II | Acute respiratory distress syndrome | Days alive and free of organ support to day 28 | Acetaminophen decreases the mortality |
| Acetaminophen |  | NCT01120769 | NA | Reperfusion injury in acute myocardial infarction | Plasma isoprostane level | NA |
| Auranofin | Inhibiting GSH biosynthesis | NCT02770378 | I/II | Glioblastoma | Toxicity, tumor response | Auranofin displays a strong anti-proliferative and anti-migratory activity |
| Auranofin |  | NCT01737502 | I/II | Recurrent non-small cell lung  cancer or small cell lung cancer | Adverse events, progression-free survival rate | Ongoing |
| Auranofin |  | NCT01419691 | II | Chronic lymphocytic leukemia | Response rate, adverse events | Ongoing |
| Auranofin |  | NCT01747798 | I | Recurrent epithelial ovarian; primary peritoneal, or fallopian tube cancer | The expression of CA125 | Ongoing |
| NAC | GSH synthesis regulator | NCT04481048 | II | Neurofibromatosis Type 1 | Change from Baseline in Motor Function | Ongoing |
| NAC |  | NCT04081012 | NA | Chronic thromboembolic  pulmonary hypertension | Presence of post-reperfusion pulmonary injury. | Ongoing |
| NAC |  | NCT03306979 | II | Vascular cognitive impairment  no dementia | Change in executive function | Ongoing |
| NAC |  | NCT03493178 | I | Mild cognitive impairment | Cognition | Ongoing |
| Baicalein | ALOX12 inhibitor;  ALOX12/ALOX15  inhibitor; ACSL4  inhibitor | NCT03830684 | II | Influenza | The time of fever relieving | NA |
| Nordihydroguaiaretic acid (NDGA) | Pan-LOX inhibitor | NCT00678015 | II | Prostate cancer | Prostate specific antigen (PSA) Response | NDGA therapy lengthens median PSADT but does not induce significant PSA declines. |
| Pioglitazone | Inhibition of ACSL4 | NCT05013255 | II | Breast cancer | Muscle Gene Expression | Ongoning |
| Rosiglitazone |  | NCT04114136 | II | Solid tumor malignancies | Best overall response | Ongoing |
| Rosiglitazone |  | NCT00182052 | III | Prostate cancer | Changes in PSA | Rosiglitazone did not increase PSADT or prolong the time to disease progression more than placebo |
| Rosiglitazone |  | NCT00688207 | I | Alzheimer’s disease | Peak concentration | NA |
| Troglitazone |  | NCT00003058 | II | Sarcoma | NA | Terminal adipocytic differentiation was induced in these malignant tumors by troglitazone |
| Zileuton | ALOX5 selective  inhibitor | NCT02047149 | I | Chronic myelogenous leukemia | Maximal tolerated dose | NA |
| Zileuton |  | NCT01130688 | I | Chronic myelogenous leukemia | Safety | No clinically significant change in the profile of adverse events |
| Zileuton |  | NCT00056004 | II | Lung cancer | Bronchial dysplasia number and grade at 6 months | NA |
| Zileuton |  | NCT00070486 | II | Lung cancer | Disease free survival | Failed to demonstrate the therapeutic benefit |
| Resveratrol | Peroxidases inhibition | NCT02219906 | NA | Metabolic syndrome | Platelet activation, platelet oxidative stress, platelet oxidation levels, serum thromboxane | NA |

Abbreviation: ACSL4, acyl-CoA synthetase long-chain family member 4; ALOX, arachidonate lipoxygenase; BSO, buthionine sulfoximine; HMGCR, 3-Hydroxy-3-Methylglutaryl-CoA Reductase; SLC7A11, NA, not acquired; NAC, N-Acetyl-L-cysteine; Solute Carrier Family 7 Member 11.
